# Colorimetric sensor arrays for the differentiation of baijiu based on amino-acid-modified gold nanoparticles

**DOI:** 10.1038/s41598-022-21234-z

**Published:** 2022-11-03

**Authors:** Junjie Jia, Suyi Zhang, Long Ma, Lei Zheng, Songbai Yu, Caihong Shen, Haiyan Fu, Songtao Wang, Yuanbin She

**Affiliations:** 1 Luzhou Pinchuang Technology Co. Ltd., Luzhou, 646000 People’s Republic of China; 2 National Engineering Research Center of Solid-State Brewing, Luzhou Laojiao Co. Ltd., Luzhou, 646000 People’s Republic of China; 3 College of Chemical Engineering, Zhejiang University of Technology, Hangzhou, 310032 People’s Republic of China; 4 College of Pharmacy, South-Central University for Nationalities, Wuhan, 430074 People’s Republic of China

**Keywords:** Optical spectroscopy, Sensors

## Abstract

It is of great significance for quality control to realize the discrimination for baijiu from different brands and origins. Strong-aroma-type baijiu (SAB), one of the most important Chinese aroma-type baijiu, exhibits the largest variety and market share. In this study, we proposed colorimetric sensor arrays based on gold nanoparticles (AuNPs) modified with different amino acids (AAs) to recognize the organic acids, and further distinguish different SABs. Three representative AAs, namely methionine (Met), tryptophan (Trp), and histidine (His), were selected to modify the AuNPs surface. The investigation of the effect of the main ingredients of SAB on AA@AuNPs aggregation confirmed that this aggregation mainly resulted from organic acids. Moreover, this aggregation was successfully used for differentiating 11 organic acids. Different pH conditions can not only cause changes of the content of organic acids in baijiu, but also disrupt the balance among flavor substances of baijiu to some extent. Consequently, the AA@AuNPs arrays under two pH conditions have been successfully applied to distinguish 14 kinds of SABs from different brands and origins. The proposed colorimetric sensor method is simple, rapid, and visualized and provides a potential application prospect for the quality control of baijiu and other alcoholic beverages.

## Introduction

Baijiu, one of the oldest distilled liquors in the world with about two thousand years, has a unique special flavor and taste because of its distinctive production process. The presence of > 2000 trace compounds, including esters, alcohols, acids, aldehydes, ketones, and acetones, has been identified in baijiu^1^. There are 12 kinds of aroma types on baijiu, resulting in a wide variety of baijiu products. Strong-aroma-type baijiu (SAB), also known as Luzhou-flavor baijiu, is one of the most popular baijiu in China and accounts for > 70% of the market^2^. SAB is produced from either only sorghum or a mixture of corn, rice, wheat, peas, millet, and sorghum and is mixed with a fermenting agent called “Daqu” for simultaneous saccharification and fermentation. Then, this mixture is distilled, and raw baijiu is obtained. It is a cycle fermentation process that occurs in aging mud cellars, which are rich in microorganisms^3^. SAB is widely distributed in various provinces, among which Sichuan, Anhui, and Jiangsu are the main origins. Similar production processes lead to similar flavor characteristics in different SAB. This renders the quality control of SAB considerably difficult. Therefore, distinguishing different brands and grades of SAB for quality control is crucial.

Currently, total acids, total esters, and individual characteristic components (e.g., ethyl caproate) are used as the main markers to evaluate the quality of baijiu. More trace components are required for detection by combining multiple pre-treatment methods, such as liquid–liquid extraction/liquid–liquid microextraction (LLE/LLME), headspace solid-phase microextraction (HS-SPME), and stir bar sorptive extraction (SBSE), with detection techniques, including gas chromatography-mass spectrometry (GC–MS), gas phase ion migration spectrometry (GCIMS), liquid chromatography-mass spectrometry (LC–MS)^1,4^. Most of these methods involve expensive equipment and complex, time-consuming, and laborious operating procedures. It is difficult to realize real-time monitoring of the baijiu brewing process, which seriously impedes quality improvement and technological innovation of baijiu. Therefore, it is extremely urgent to develop a method for fast detection of baijiu quality.

The operations of spectroscopic techniques and commercial electronic noses are simple and fast. However, their detection sensitivity and resolution are relatively low for the components of baijiu, which makes the classification of baijiu samples with similar flavor difficult^5–7^. In recent years, some sensing detection methods, such as electrochemical impedance spectroscopy based on graphite electrodes^8^, colorimetric sensing method using organic dye molecules as sensitive materials, and fluorescence sensing method utilizing conjugated polymers as fluorescence materials^9–12^, have been employed for wine and liquor identification. These new sensing methods have substantially simplified operation procedures and improved sensitivity; however, problems, such as relatively low stability and selectivity, have not been well solved.

Compared with traditional single sensors, sensor arrays present the potential to identify multiple targets spontaneously. Additionally, sensors can recognize subtle differences in similar chemical structures among analytes. Colorimetric sensor arrays have received attention in many sensing applications because of their advantages, such as fast analyses, low cost, easy use, and visual results^13^. The unique local surface plasmon resonance (LSPR) properties of gold nanoparticles (AuNPs) make them excellent sensing materials for the preparation of novel chemical and biological sensors and are widely employed for detecting and recognizing various components, such as metal ions, small organic molecules, biological macromolecules, microorganisms, and cells^14,15^. AuNPs exhibit tunable color changes with morphologies, such as shape, size, interparticle distance, and surrounding environment, because of LSPR. The rapid synthesis of AuNPs not only ensures tunable size and shape, but the AuNPs surface can be easily functionalized by various ligands such as thiols and amino groups^16^. Within all these excellent properties, AuNPs show great potential for colorimetric sensor array development for the detection of multiple analytes. Presently, they have been used for multi-component detection and identification because of their excellent optical sensing properties. Such as, based on the gold nanoparticles (AuNPs) aggregation effect, AuNPs can be used to recognize different organophosphorus pesticides under various conditions^17^; AuNPs are modified with different charged groups to identify different microorganisms^18^, and functionalized AuNPs and silver nanoparticles (AgNPs) are utilized to recognize gaseous molecules^19,20^. Depositing different thicknesses of silver shells on the gold nanorods (AuNRs) surface makes AuNRs exhibit distinctive multicolor changes, which are used to distinguish various catechol isomers^21^. Based on the etching effect, Ag@AuNPs and TriAgNPs have been employed for the identification of different oxidizing anions and antioxidants, respectively^22,23^. In recent years, it has been reported to distinguish different types of baijiu based on AuNPs aggregation effect, AuNRs silver deposition, and the AgNPRs etching effect^24–26^. The colorimetric sensing strategy based on the aggregation mechanism is simpler, more stable, and easier to use than that based on the other two mechanisms.

In this study, we developed a colorimetric sensor based on AuNPs modified with different amino acids (AAs) and studied the response to the main components of baijiu. By changing the pH conditions of the detection system, the expanded sensor arrays were employed to discriminate different SAB samples, which further enriches the construction strategy of colorimetric array based on AuNPs for recognizing baijiu. The response data were analyzed using linear discriminant analysis (LDA).

## Experimental

### Material and reagent

Hydrogen tetrachloroaurate (HAuCl_4_·3H_2_O), sodium citrate, L-methionine, L-tryptophan, L-histidine were purchased from J&K Chemical Ltd. (Beijing, China). Ethanol, acetic acid, propanoic acid, lactic acid, butanoic acid, isobutanoic acid, valeric acid, isovaleric acid, hexanoic acid, heptanoic acid, octanoic acid, benzoic acid, 1-propanol, isobutanol, isoamylol, acetaldehyde, acetal, ethyl acetate, ethyl butyrate, ethyl lactate, ethyl hexanoate, and ethyl heptanoate were purchased from Energy Chemical Co. Ltd. (Shanghai, China). All chemicals were of analytical reagent grade. All the flavor compound solutions were prepared with 52% ethanol.

LZ1–LZ8 were provided by Luzhou Laojiao Co. Ltd. (Luzhou, China), and other SAB samples were purchased from specialty stores in China. Milli-Q water (18.2 MΩ cm^−1^) was used for all the experiments.

Transparent 96-well microplates (Costar 3599) were purchased from Corning Incorporated.

### Instrumentation

Absorbance spectra were recorded using a Hitachi U3900 spectrophotometer. pH was measured using a REX PHSJ-3F pH meter equipped with a glass electrode. Nanoparticles (NPs) were characterized through transmission electron microscopy (TEM) (Jeol 2100F). Zeta potential was measured using Malvern Zetasizer Nano ZS90. Raman spectra were recorded using LabRAM HR UV 800 (Jobin Yvon, France). All images were acquired with a digital camera (Canon 750D).

### Synthesis of AA@AuNPs

Citrate-capped AuNPs were prepared using the method described^17^. First, 5 mL of 38.8 mM trisodium citrate was added to the boiling HAuCl_4_ solution (1 mM, 50 mL) with vigorous stirring. Then, the resulting mixture was heated under reflux for 30 min. During the heating process, the mixture color changed to deep red, indicating the formation of AuNPs. The solution was allowed to cool at room temperature and stored at 4 °C for further use.

AA@AuNPs were prepared by referring to the methods reported in literature as follows^27–29^: 20 μL of 10 mM AA solution was added to 50 mL of AuNPs with stirring. After 2-h stirring at 30 °C, AA@AuNPs were prepared. In order to obtain the pure AA@AuNPs solutions, the remaining amino acids unmodified on the surface of AuNPs were removed by ultrafiltration, which was performed using commercialized ultrafiltration centrifuge tubes (10 KD). AA@AuNPs solutions were centrifuged at 5000 rpm for 10 min, and then, unfiltered deposits were resuspended using the same amount of deionized water for further use.

### Sensor array fabrication and sample detection

In this study, pH values were adjusted by adding different concentrations of HCl or NaOH solution to the reaction system. Consider the pH of 6.5 and 10.5 as examples. The specific adjustment followed methods are as follows. For the colorimetric detection system at the pH of 6.5, 70 μL of H_2_O was added to 67.5 μL of AA@AuNPs, and then, mixed with 12.5 μL of 50 mM flavor compound solution or baijiu samples. For the colorimetric detection system at the pH of 10.5, 56.5 μL of H_2_O and 13.5 μL of 50 mM NaOH solution were added to 67.5 μL of AA@AuNPs. Then 12.5 μL of the baijiu sample was added to this solution. The detection system was magnified 10 times for UV–Vis detection. All colorimetric experiments were performed in triplicate on 96-well plates.

### Data analysis

The images and UV–Vis spectra were obtained after the color response of 5 min. Linear discriminant analyses were performed on a data matrix by using SYSTAT (version 13.2). The operation and instructions for this software are reported in the literature^26^. PLSR calibration and prediction were performed with Camo’s Unscrambler software (The Unscrambler X 10.4).

## Results and discussion

### Sensor array design and fabrication

**Figure 1:**
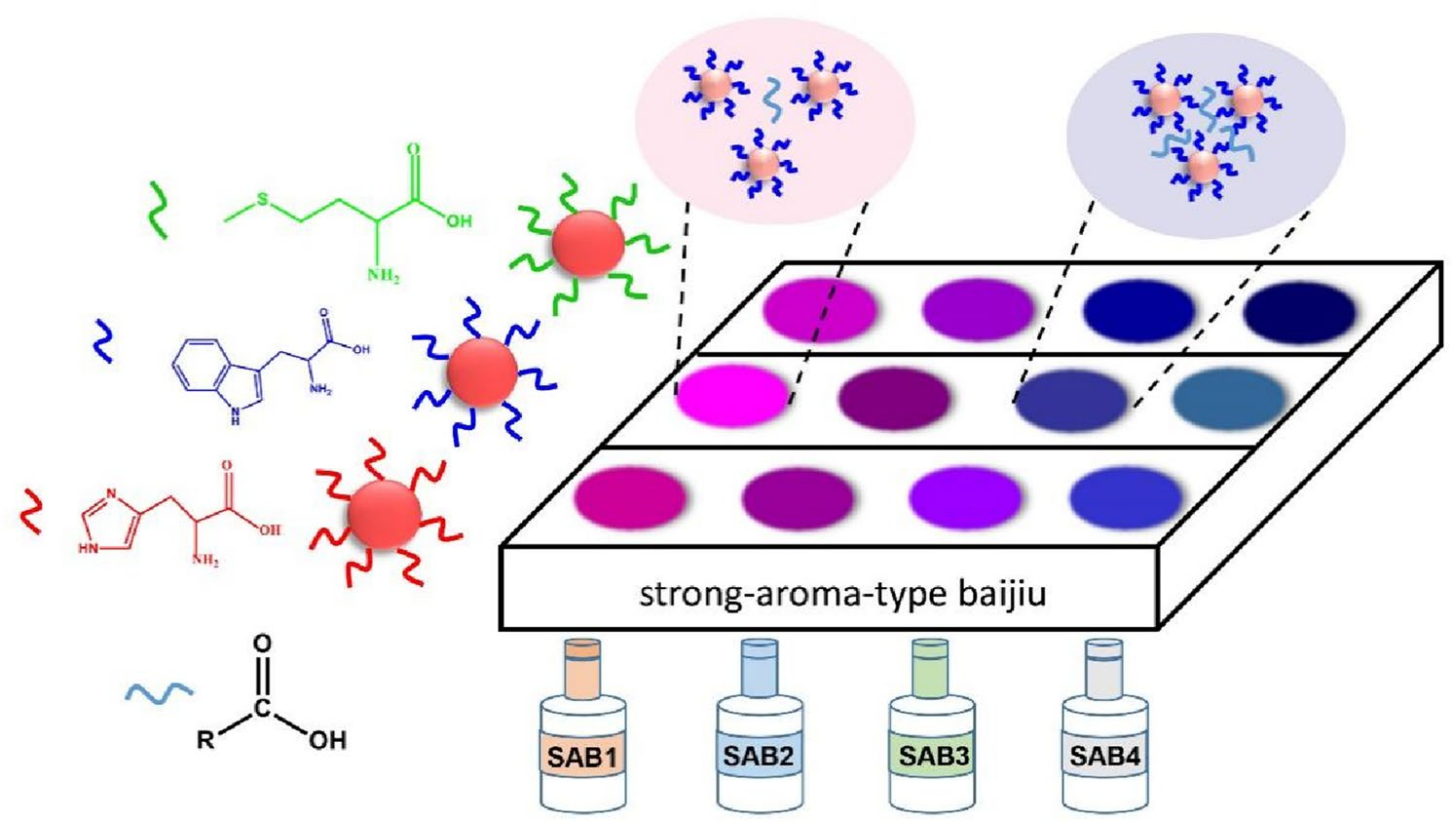
Colorimetric sensor array based on AA-modified AuNPs for recognition of Chinese SAB.

Small molecules, such as amino acids and sulfhydryl compounds, are more easily and controllably modified on the surface of AuNPs due to the less complex structure and steric hindrance, which make them widely applied for the development of biochemical sensors^14^. AuNPs modified with diverse small molecules show different binding properties with various analytes. Baijiu comprises rich flavor compounds, such as alcohols, organic acids, esters, and aldehydes. Among them, organic acids containing carboxyl groups serve as ester precursors in baijiu and are the most important taste-sensitive compounds^30^. Consequently, inducing AA@AuNPs aggregation through hydrogen bonds or electrostatic interactions between the AAs and carboxyl group of organic acids is easy and accompanied with various color changes (Fig. 1). Therefore, we developed a colorimetric sensor array based on AuNPs modified with different amino acids in order to recognize different various organic acids and to further distinguish different baijiu samples.

In this study, three representative AAs, namely sulfur-containing AAs (Met), polar AAs (Trp), and basic AAs (His), were selected as modification molecules. These three AAs show considerable differences in their structures and properties, indicating that they may induce different degrees of AA@AuNPs aggregation in the presence of baijiu. The syntheses of AA-modified AuNPs have been reported in the literature. Most of these synthesis methods are based on thermal reduction. However, AA@AuNPs synthesized using this method are not stable and easily lead to AA@AuNP aggregation^31,32^. In this study, sodium citrate modified AuNPs (Cit@AuNPs) were used as the initial substrate and were functionalized by mixing them with AAs at room temperature, which ensured the stability and consistency of particle size.

**Figure 2:**
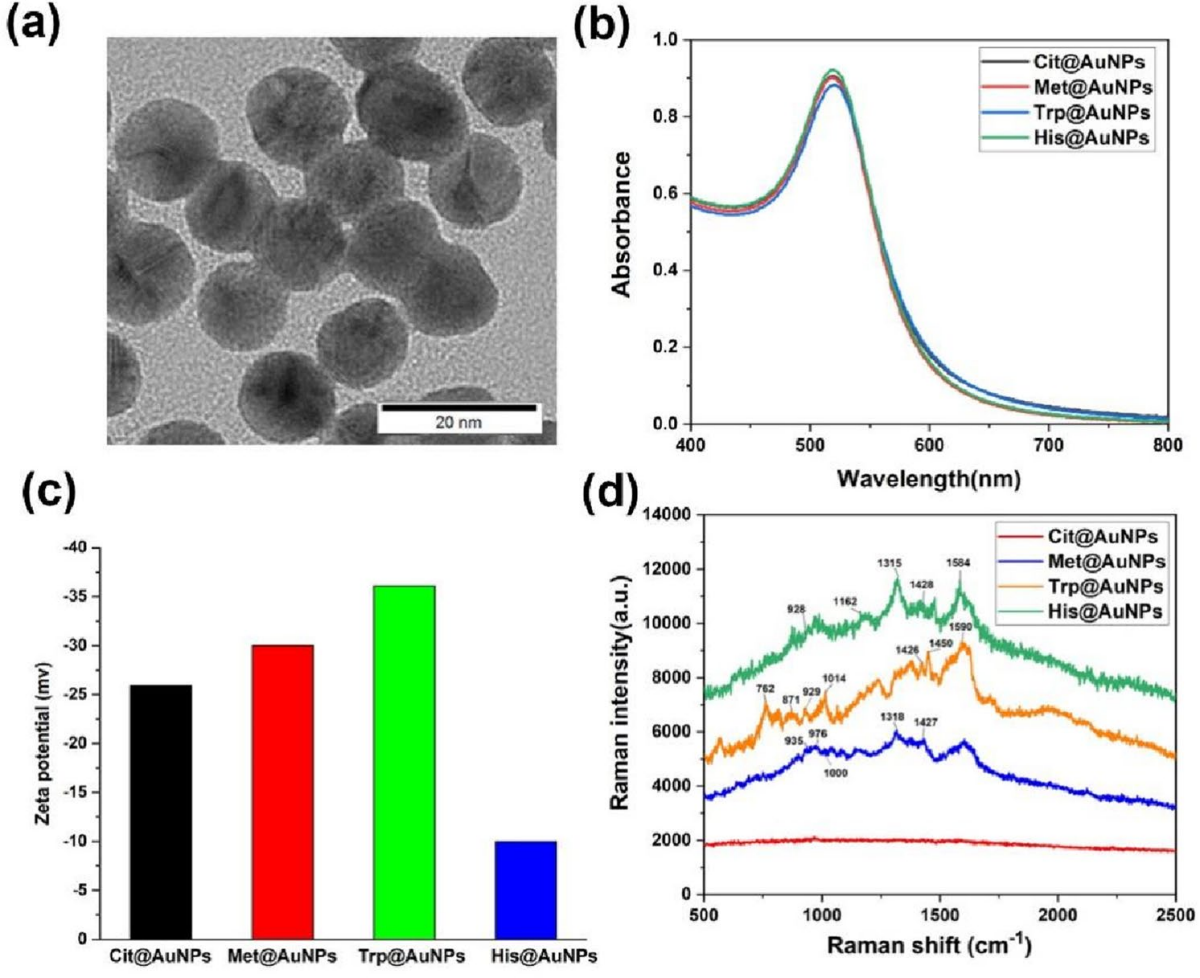
(**a**) TEM image of as-prepared Cit@AuNPs, (**b**) UV–Vis spectrum, (**c**) Zeta potentials, and (**d**) Raman spectra of AA@AuNPs.

First, we optimized the AA modification concentration. The optimized AA concentration was the minimum concentration that did not cause Cit@AuNPs aggregation. Then, unmodified AAs were removed through ultrafiltration. The AA modification concentration of 5 µM was selected (Fig. S1). The three synthesized AA@AuNPs were characterized through TEM, UV–Vis, Zeta potential, and Raman spectroscopy (Fig. 2). Figure 2a shows that the AuNPs size is uniform with an average particle size of 12 nm (Fig. S2), and Fig. S3 illustrates that AA modification did not affect the nanoparticle morphology. Figure 2b shows that LSPR peaks for Met@AuNPs, Trp@AuNPs, and His@AuNPs are acquired at 518, 520, and 519 nm, respectively. Compared with Cit@AuNPs, AA@AuNPs exhibited no potential changes. Met@AuNPs and Trp@AuNPs showed a more negative charge, whereas His@AuNPs had a less negative charge, which indicated that AA were modified on the AuNP surface (Fig. 2c). Met was bound to the AuNPs surface mainly through Au–S bonding; Trp was bound to the AuNPs surface mainly through amino and indole groups; and the carboxyl group was exposed^33^. His@AuNPs with less negative charge may correspond to the imidazole group, which is consistent with the reported results that amino acids were bound to the surface of AuNPs through the α-amino group, and the carboxyl group was outward^34^. Raman spectroscopy can directly measure and characterize liquid samples, usually requires no sample preparation, and acquire information about molecular structure, this method is simple and fast. Furthermore, Raman spectroscopy has been reported for the characterization of nanoparticle surface groups^27^. Raman spectroscopy was employed to characterize the modified groups of AA@AuNPs (Fig. 2d). AA@AuNPs show the peak centered at 1427 (1426, 1428) cm^−1^, which can be assigned to COO– symmetric stretching vibrations, and the peaks around 929 (928, 935) cm^−1^ correspond to C–COO– stretching vibrations. For His@AuNPs, the strong bands obtained at 1584, 1315, and 1169 cm^−1^ were assigned to C–C stretching vibrations, CH_2_ wagging vibrations, and the stretching of the C=N bond of the imidazole ring, respectively^35,36^. The peaks seen at 764 and 871 cm^−1^ are assigned to a normal mode combining the CH bending of the six-membered ring and the in-phase breathing of both the five and six-membered rings in Trp@AuNPs, respectively^37–39^. Another three intense peaks appearing at 1014, 1450, and 1590 cm^−1^ correspond to the twisting vibration of the CH_2_ group, scissoring vibration of the CH_2_ group, and C–C stretching of the five-membered ring, respectively^40^. The two small bands situated at 976 and 1000 cm^−1^ can be attributed to the methionine side chain. Moreover, the peak appearing at 1318 cm^−1^ is associated with the twisting of the CH_2_ group^40^. The considerable increase in the peaks indicated the AAs were bound to the AuNPs surface.

### Effects of the main elements of baijiu

**Figure 3:**
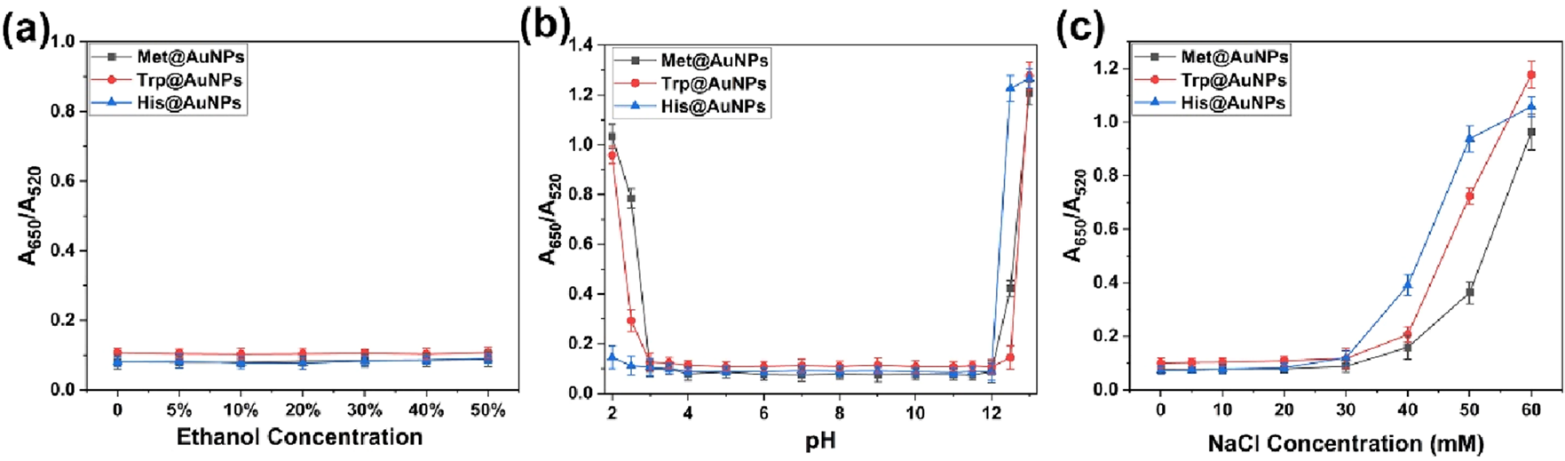
Effects of (**a**) ethanol concentration, (**b**) pH, and (**c**) NaCl concentration on the stability of AA@AuNPs.

Ethanol and water make up 98–99% components of baijiu. Thus, we studied the effects of different concentrations of ethanol on the stability of AA@AuNPs. When the ethanol content increases to 50% volume in the reaction system, the absorption peaks of three AA@AuNPs and their corresponding colors show no change (Fig. 3a). In the detection system of 150 µL, the additional amount of baijiu is only 12.5 µL; that is, the amount of ethanol in the system is < 5% volume. Thus, the ethanol content in baijiu could not cause AA@AuNPs aggregation in the system.

As previously reported, the electrolyte solution and ionic strength may induce a certain degree of aggregation of AuNPs^17,41,42^. To avoid the introduction of more complex electrolytes with the general pH buffers, we adjusted pH by adding a certain concentration of NaOH or HCl solution, which only introduced Na^+^ or Cl^−^ into the reaction system. This strategy for adjusting pH in the sensor system based on AuNPs has been reported^17,33^. Therefore, we further studied the effect of the NaCl concentration on AuNPs aggregation. Due to the presence of organic acids, the pH of baijiu is usually between 3 and 5. The pH of SAB in this paper is 3.5–4 (Table S1). The effect of pH on AA@AuNP aggregation was studied. AA@AuNPs are not affected by pH when the pH of the reaction system is 3–12 (Fig. 3b). When the pH was < 3 or > 12, AA@AuNPs aggregated to different degrees. In our experimental system, the additional amount of baijiu accounted for < 10%; thus, the pH value was between 3 and 7. We further confirmed that the NaCl concentration of < 30 mM does not cause AA@AuNPs aggregation (Fig. 3c), while the concentration of Na^+^ and Cl^-^ introduced by adjusting the pH in the detection system was much lower than 30 mM. Therefore, the sensor detection system under pH 3–12 conditions did not cause AA@ AuNPs aggregation.

### Recognition of organic acids

**Figure 4:**
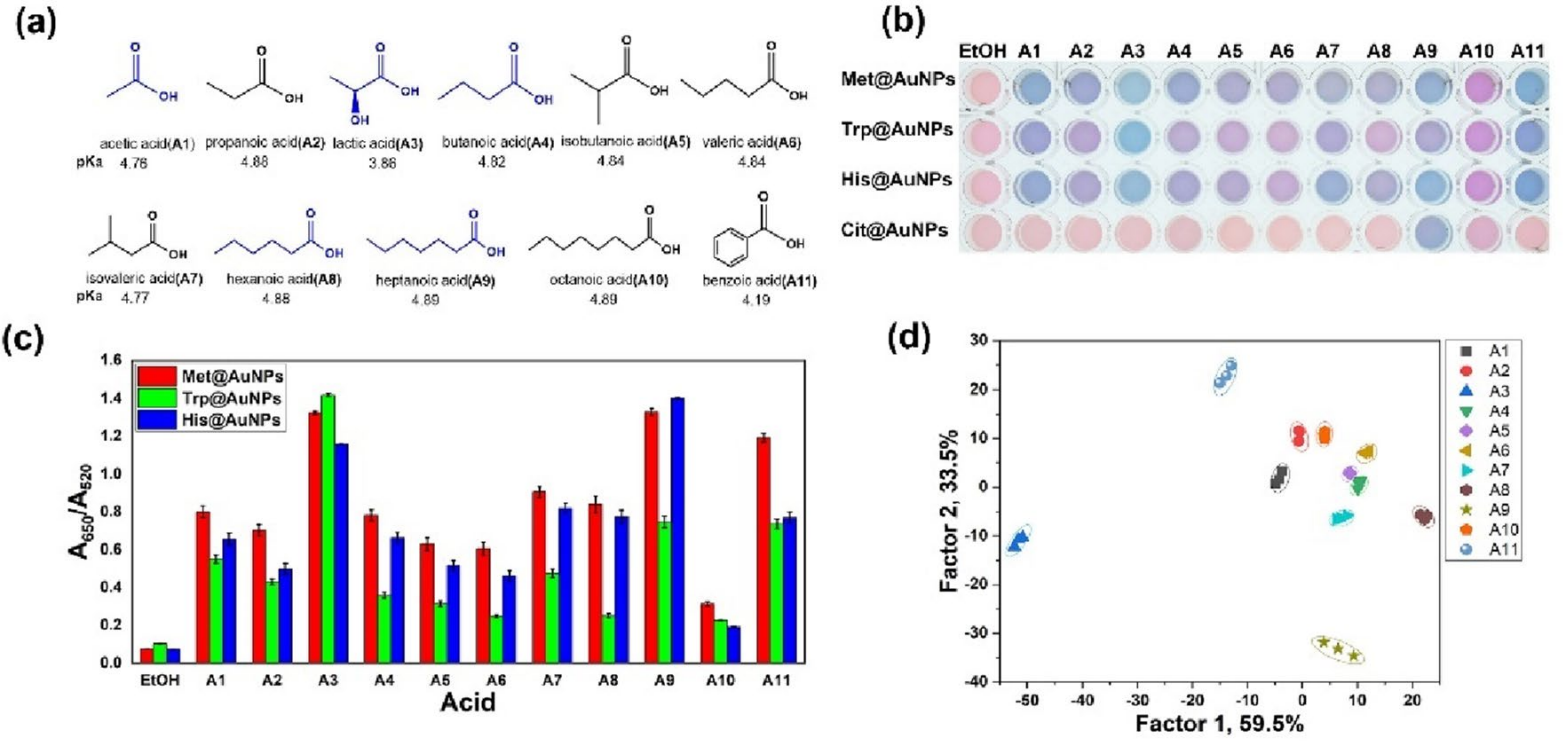
(**a**) Molecular structures of main organic acids in SAB, (**b**) color change patterns, (**c**) response (A650/A520) patterns, and (**d**) two-dimensional canonical score plots for the first two factors of the 11 organic acids (0.5 g/L) obtained using the sensor array based on AA@AuNPs.

Organic acids are the most important taste-sensitive compounds in baijiu and are the precursor of esters, which can eliminate bitter taste in baijiu and promote the aging of fresh baijiu. In general, in the production of Chinese baijiu, organic acids are evaluated to monitor the whole production process and ensure product quality^30^. Due to the presence of carboxyl groups, it is easy to interact with AA@AuNPs through hydrogen bond or electrostatic interaction for the organic acids, which results in AA@AuNPs aggregation and LSPR changes. To verify this mechanism, 11 types of organic acids, the most vital taste compounds of SAB^30,43^, were selected to study their influence on AA@AuNPs aggregation (Fig. 4a). Acetic acid (A1), lactic acid (A3), butyric acid (A4), and caproic acid (A8) are the most crucial organic acids in SAB^1^. Additionally, heptanoic acid (A9) is an important flavor compound for SAB^43^. Five organic acids (blue in Fig. 4a) account for > 90% of the total organic acids in SAB. The 11 organic acids lead to varying degrees of AA@AuNPs aggregation, which indicated Cit@AuNPs had no obvious response to other organic acids except heptanoic acid, and its response to heptanoic acid was not as strong as Met@AuNPs and His@AuNPs (Fig. 4b). The color changes of AA@AuNPs were also recorded by UV–Vis absorption spectra (Fig. S4). Colorimetric response was obtained using A650/A520 and was utilized to evaluate AA@AuNPs aggregation. The color change is evident for lactic acid, heptanoic acid, and benzoic acid, and A650/A520 values are high (Fig. 4c). The colorimetric response of propionic, butyric, isobutyric, valeric, isovaleric, and caproic acids was relatively weak, indicating a low response value, which is consistent with the results presented in Fig. 4b. Finally, the different response values (A650/A520) were analyzed by conducting LDA, which reduced the size of the training matrix (3 sensing elements × 11 acids × 3 replicates) and transformed them into canonical factors. The canonical score plot for the first two factors was obtained using the sensor arrays for the 11 types of organic acids presented in Fig. 4d, and the jackknifed classification matrix revealed 100% accuracy through cross-validation. Acetic, lactic, butyric, and caproic acids are the most vital four organic acids for SAB, which account for > 90% of total organic acids. The colorimetric sensor array based on AA@AuNPs provides considerable discrimination capacity for the four organic acids, especially lactic acid is the most responsive because of its unique structure (Fig. 4d).

In addition, we studied the response of the array to some crucial esters (e.g., ethyl acetate, ethyl butyrate, ethyl lactate, ethyl hexanoate, and ethyl heptanoate), alcohols (e.g., propanol, isobutanol, and isoamylol), and aldehydes (acetaldehyde and acetal) with the same concentration. These esters, alcohols, and aldehydes are important aromatic compounds in SAB. The results showed no obvious response (Fig. S5). This finding further confided that AA@AuNPs mainly interacted with organic acids, resulting in AA@AuNPs aggregation.

To further evaluate the quantitative ability of the colorimetric sensor array, the heptanoic acid with good response was selected for quantitative by PLSR model (Fig. S6). The quantitative range of heptanoic acid was set at 0.1–1.0 g/L as the model training set, and a relatively sufficient number of heptanoic acid concentration samples, such as 0.15–0.95 g/L, were selected as the prediction set of the model by referring to the partition ratio between the prediction set and the training set reported by Tian et al^44^. The quantitative results are shown in Table S2, and indicated a good linear relationship between the colorimetric change of the sensor array and the concentration of heptanoic acid.

### Discrimination for SAB

The aforementioned studies have confirmed that organic acids, as the most important taste-sensitive compounds in baijiu (especially in SAB), can induce different degrees of AA@AuNPs aggregation. Therefore, we considered that the array can be used to evaluate the differentiation performance of SABs from different brands and origins. SAB comprises highly complex flavor compounds, including organic acids, esters, and alcohols, which account for > 90% of the contents of all flavor ingredients. Organic acids and alcohols are precursors for esters, which can be reversibly hydrolyzed into acids and alcohols under acidic or alkaline conditions. Therefore, it presents a dynamic balance among the three components. Moreover, organic acids can undergo hydrolysis to different degrees under different acidic or alkaline conditions. Therefore, pH changes in the reaction system can not only cause variations in organic acid content but also affect the balance among the three components in the presence of baijiu. Accordingly, the contents of the flavor components in the baijiu samples changed under different pH conditions, especially the content of organic acids.

The response of AA@AuNPs colorimetric sensor arrays was related to baijiu varieties, detection system under different pH conditions and the structural characteristic of AA@AuNPs. It is acidic (pH 3.4–4.0) due to a variety of organic acids for SAB. The aggregation kinetics are faster at lower (acidic) pH values, which attribute to the effect of protonating the carboxyl groups^33^. When SAB samples were added to the detection system at low pH values, different SAB samples caused different degrees of protonation, resulting in different degrees of AA@AuNPs aggregation. However, the dissociation of organic acids is inhibited under acidic conditions, resulting in lower ionic strength, which could resist AA@AuNPs aggregation to a certain extent. With the increase of pH value, when the detection system was in alkaline condition and the organic acids of SAB samples were gradually neutralized, the degree of protonation gradually weakened, resulting in the degree of AA@AuNPs aggregation gradually weakened. Due to positively charged imidazole group for His@AuNPs, makes them less negatively charged than Met@AuNPs and Trp@AuNPs, resulting in relatively obvious aggregation than other two AA@AuNPs (Fig. S7). However, when the pH value gradually increased, such as pH 11.5, the alkaline condition became the main factor affecting the stability of AA@AuNPs, acid–base neutralization made baijiu samples insufficient to cause AA@AuNPs aggregation.

**Figure 5:**
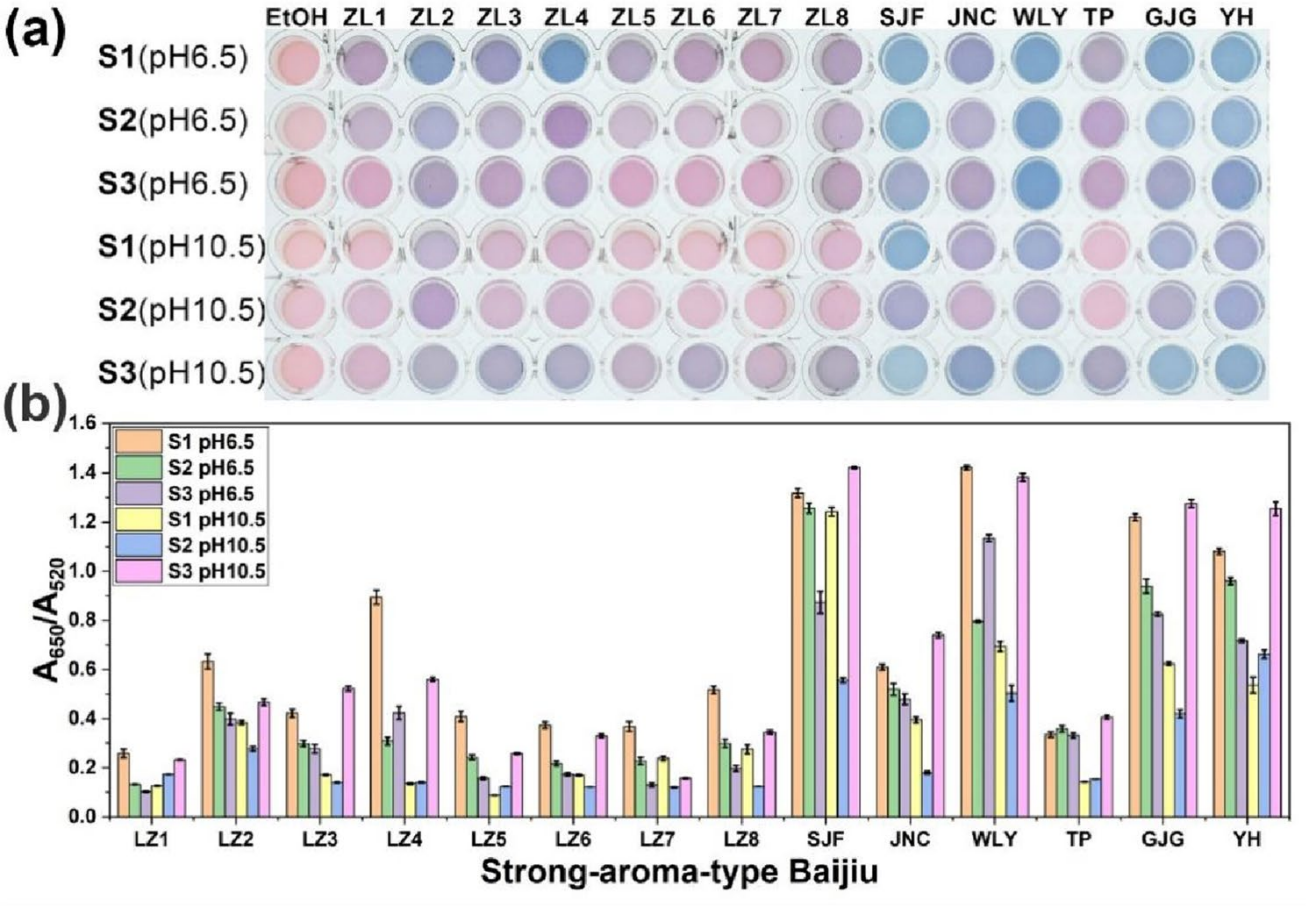
(**a**) Color change patterns and (**b**) response (A650/A520) patterns of 14 kinds of SAB samples obtained using the sensor array based on AA@AuNPs.

Furthermore, the recognition performance of the sensor array under three representative pH conditions for 14 SABs was evaluated by LDA. The results showed that the relatively low discrimination accuracy of 93% and 66% under pH 3.5 for different brands and different origins of SAB, respectively (Fig. S8). Therefore, the responses at pH 6.5 and pH 10.5 were performed LDA analysis. The array provides different colorimetric responses to different SAB samples under the two pH conditions (Fig. 5a). The color change was also recorded using UV–Vis absorption spectra (Fig. S9). A650/A520 was used to evaluate AA@AuNPs aggregation caused by different SABs (Fig. 5b). The findings are consistent with the results presented in Fig. 5a.

**Figure 6:**
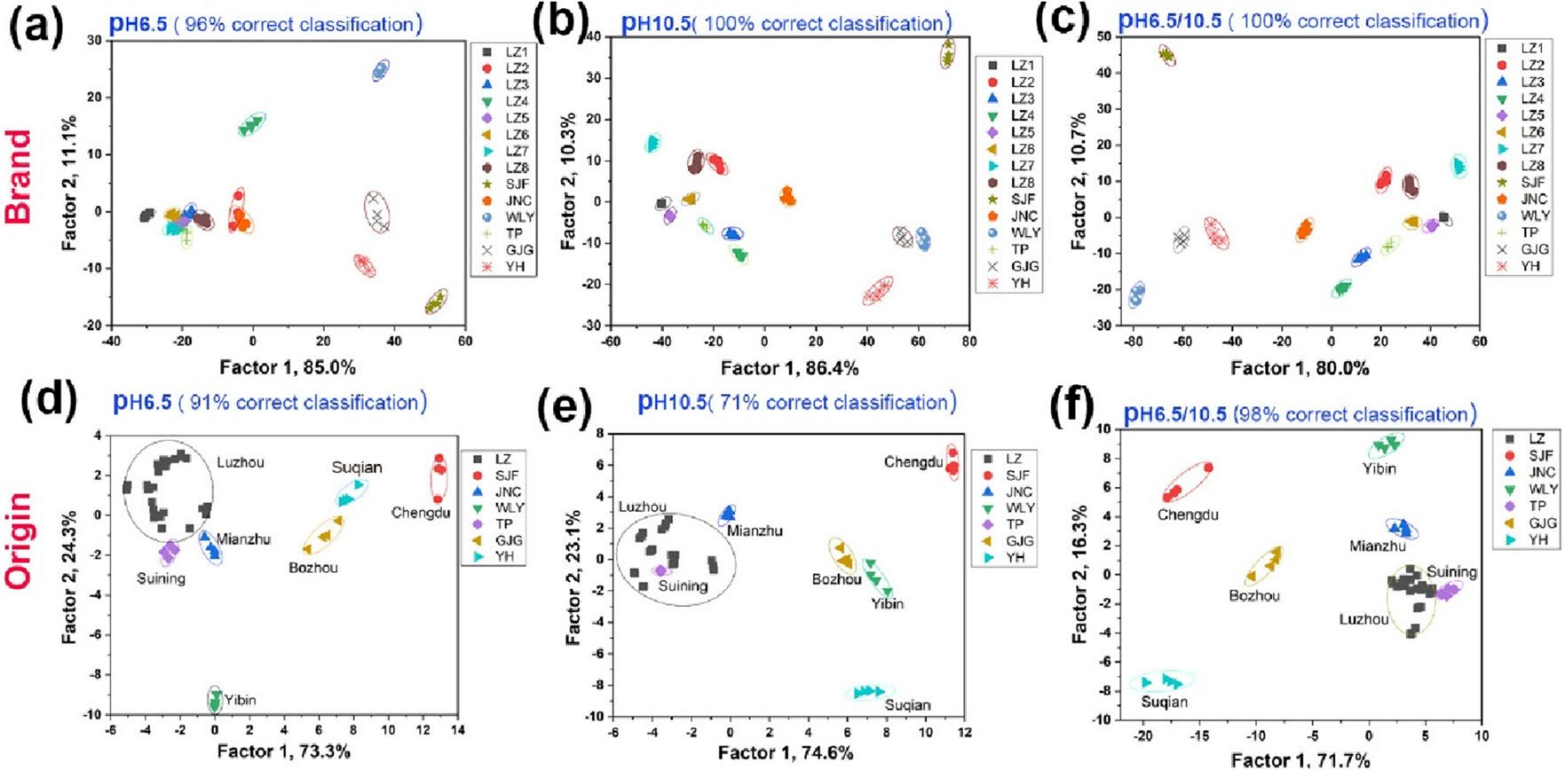
Two-dimensional canonical score plots for the first two factors of (**a**–**c**) 14 brands and (**d**–**f**) 7 origins obtained using the sensor array based on AA@AuNPs at the pH of (**a**, **d**) 6.5, (**b**, **e**) 10.5, and (**c**, **f**) 6.5 and pH 10.5.

Different response values under the two pH conditions were used to evaluate the distinguishing performance for SABs from different brands and origins by LDA (Fig. 6a). The array cannot be used to well distinguish all the 14 SABs, especially Luzhou SABs with different grades at the pH of 6.5 (Fig. 6a). However, all the 14 SABs can be well distinguished at the pH of 10.5 (Fig. 6b). When the arrays were combined under the two pH conditions (Fig. 6c), the differentiation of 14 SABs was similar to that presented in Fig. 6b. The jackknifed classification matrix with cross-validation revealed 100% accuracy for the arrays at the pH of 10.5 and under the two conditions (Tables S3–S5, Figs. S10–S12).

**Figure 7:**
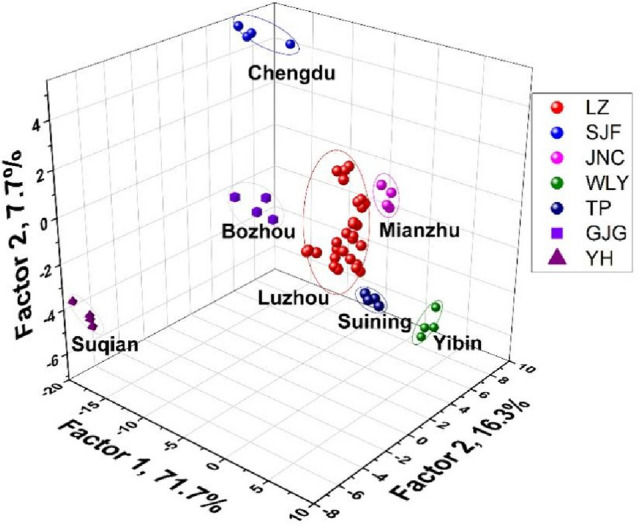
3D canonical score plot for the first three factors of 7 origins obtained using the sensor array based on AA@AuNPs under the two pH conditions.

Sichuan, with unique regional features that cannot be duplicated, is an important province for SAB production. It provides popular baijiu brands, such as Wuliangye, Luzhou Laojiao, Jiannanchun, Tuopai, and Shuijingfang. Bozhou, Anhui, and Suqian, Jiangsu, are the other two representative SAB-producing areas. Hence, differentiating the varieties of SAB samples according to their origins is vital. As shown in Fig. 6d, the jackknifed classification matrix with cross-validation of the array for the differentiation of different origins at pH 6.5 was 91%, among which four Luzhou SABs were wrongly judged as Jiannanchun and one as Tuopai (Table S6, Fig. S13). The cross-validation accuracy was only 71% at the pH of 10.5 (Table S7, Fig. S14), and 50% of Luzhou SABs were misjudged as Tuopai SABs (Fig. 6e). When the arrays with the two pH conditions were combined, the distinguishing performance was significantly improved (Fig. 6f), and the cross-validation accuracy reached up to 98% (Table S8, Fig. 5f and Fig. S15). To show the distinction among SABs from different origins more clearly, for the first three factors of the colorimetric patterns, a 3D canonical score plot was obtained using the combined array. The array can be used to distinguish the SABs from different origins well (Fig. 7).

Furthermore, the current method and existing colorimetric sensor arrays used for baijiu identification are presented in Table 1. Compared with the existing methods, our sensor arrays based on AA@AuNPs mainly respond to organic acids in baijiu, which presents high specificity. Moreover, the material synthesis method and array expansion strategy used in this study are simple and can be employed to recognize more samples containing organic acids.

**Table 1:** Comparison of the presented method with some published baijiu colorimetric sensing methods.

Sensing material	Array points	Flavor compounds	Baijiu numbers	Analytical methods	References
Nanomaterials/dyes	9	No report	8	PCA, HCA, LDA	^46^
AgNPRs	8	No report	16	PCA, HCA, LDA, RBFN	^25^
AuNRs	12	Alcohol, aldehyde, acid, ester, ketone	24	PCA, HCA, LDA	^26^
Au TNPs	4	Acids, sulfides	16	HCA, LDA	^47^
Au NRs	4	Reducing substances	16	PCA, HCA, LDA	^48^
AuNPs	3	Acid	14	LDA	This work

## Conclusions

In this study, we developed a colorimetric sensor array based on AuNPs modified with different AAs to recognize SABs from different brands and origins. We studied the effect of the main ingredients present in SAB on the stability of AA@AuNPs (Met@AuNPs, Trp@AuNPs, and His@AuNPs) and confirmed that AA@AuNPs aggregation mainly resulted from organic acids, the most important taste-sensitive components available in SAB as the ester precursor. The sensor array can be used to distinguish organic acids. Chinese baijiu is rich in organic acids, esters, and alcohols, which show a dynamic balance, resulting in an acidic environment. Furthermore, the three types of AA@AuNPs were used to recognize the representative SAB samples under two pH conditions, and the LDA discrimination accuracy was 100%. This recognition performance can be improved by adding various AAs for AuNPs modification and using different pH conditions to increase the recognition performance of the arrays for other baijiu samples. The sensor arrays based on AA@AuNPs can also be used to recognize other aroma type baijiu samples. In addition, the response of AA@AuNPs mainly results from organic acids; thus, the sensor strategy can be applied for the identification of many beverages containing organic acids. The proposed method is simple, rapid, and visualized, which provides potential application value for the quality control of baijiu and other beverages.

## Supplementary Information

Supplementary Information.

## Data Availability

The authors declare that all data supporting the findings of this study are available within the article. Detailed information on all data presented herein are also available on request from the corresponding author Songtao Wang.
