# Supplementary material for: Colorimetric sensor arrays for the differentiation of baijiu based on amino-acid-modified gold nanoparticles
Source: Sci Rep. 2022 Nov 3;12:18596. doi: 10.1038/s41598-022-21234-z (PMC9633599; doi:10.1038/s41598-022-21234-z)
Supplement: Supplementary file 1 — Supplementary Information. [file 41598_2022_21234_MOESM1_ESM.docx]

**Supporting Information**

**Colorimetric Sensor Arrays for the Differentiation of Baijiu Based on Amino-Acid-Modified Gold Nanoparticles**

Junjie Jia,^a,b,c^ Suyi Zhang,^a,b^ Long Ma,^a,b^ Lei Zheng,^a,b^ Songbai Yu,^a,b^ Caihong Shen,^a,b^ Haiyan Fu,^d^ Songtao Wang,^*,a,b^ and Yuanbin She^*,c^

^a^ Luzhou Pinchuang Technology Co. Ltd., Luzhou 646000, PR China

^b^ National Engineering Research Center of Solid-State Brewing, Luzhou Laojiao Co. Ltd., Luzhou 646000, PR China

^c^ College of Chemical Engineering, Zhejiang University of Technology, Hangzhou 310032, PR China

^d^ College of Pharmacy, South-Central University for Nationalities, Wuhan 430074, PR China

Corresponding Author

* Yuanbin She E-mail: [sheyb@zjut.edu.cn](mailto:sheyb@zjut.edu.cn).

* Songtao Wang E-mail: lzpckj@126.com.

**Table of Contents**

**Table S1** Detailed information on the strong-aroma-type baijiu

**Figure S1**. Optimization of concentration of amino acid modified on the surface of AuNPs.

**Figure S2**. The graph of the Cit@AuNPs DLS analysis.

**Figure S3.**  The TEM of (a) Met@AuNPs; (b) Trp@AuNPs; (c) His@AuNPs.

**Figure S4.** UV-vis absorption spectra of AA@AuNPs responsed to the organic acids.

**Figure S5.** Response of the array based on AA@AuNPs to the main flavor compounds in baijiu.

**Figure S6.** UV-vis spectrum and linear relationship of His@AuNPs response to heptanoic acid of different concentrations.

**Figure S7.** Response of AA@AuNPs to five representative SABs under different pH conditions.

**Figure S8.** Two-dimensional canonical score plots for the first two factors of 14 brands and 7 origins obtained using the sensor array based on AA@AuNPs at the pH 3.5.

**Figure S9.** UV-vis absorption spectra of AA@AuNPs responses to different SABs under the two pH conditions.

**Figure S10**. Correlations of canonical colorimetric response patterns from AA@AuNPs against the different brands of SABs at pH 6.5.

**Figure S11**. Correlations of canonical colorimetric response patterns from AA@AuNPs against the different brands of SABs at pH10.5.

**Figure S12**. Correlations of canonical colorimetric response patterns from AA@AuNPs against the different brands of SABs at pH 6.5 and pH10.5.

**Figure S13**. Correlations of canonical colorimetric response patterns from AA@AuNPs against the different origins of SABs at pH 6.5.

**Figure S14**. Correlations of canonical colorimetric response patterns from AA@AuNPs against the different origins of SABs at pH10.5.

**Figure S15**. Correlations of canonical colorimetric response patterns from AA@AuNPs against the different origins of SABs at pH 6.5 and pH10.5.

**Table S2.** The results of quantitative analysis of heptanoic acid

**Table S3.** Jackknifed Classification Matrix of SABs from different brands at pH 6.5

**Table S4.** Jackknifed Classification Matrix of SABs from different brands at pH10.5

**Table S5.** Jackknifed Classification Matrix of SABs from different brands at pH 6.5 and pH10.5

**Table S6.** Jackknifed Classification Matrix of SABs from different origins at pH 6.5.

**Table S7.** Jackknifed Classification Matrix of SABs from different origins at pH10.5.

**Table S8.** Jackknifed Classification Matrix of SABs from different origins at pH 6.5 and pH10.5

**Table S1** Detailed information on the strong-aroma-type baijiu

| **Abbr.** | **Brand** | **Origin** | **pH** | **Alcohol (%v/v)** |
| --- | --- | --- | --- | --- |
| LZ-1 | Luzhou Laojiao | Sichuan Luzhou | 3.73 | 52 |
| LZ-2 | Luzhou Laojiao | Sichuan Luzhou | 3.69 | 52 |
| LZ-3 | Luzhou Laojiao | Sichuan Luzhou | 3.71 | 52 |
| LZ-4 | Luzhou Laojiao | Sichuan Luzhou | 3.80 | 52 |
| LZ-5 | Luzhou Laojiao | Sichuan Luzhou | 3.74 | 52 |
| LZ-6 | Luzhou Laojiao | Sichuan Luzhou | 3.76 | 52 |
| GJ-7 | Guojiao 1573 | Sichuan Luzhou | 3.85 | 52 |
| GJ-8 | Guojiao 1573 | Sichuan Luzhou | 3.89 | 52 |
| SJF | Shuijingfang | Sichuan Chengdu | 3.89 | 52 |
| JNC | Jiannanchun | Sichuan Mianzhu | 3.66 | 52 |
| WLY | Wuliangye | Sichuan Yibin | 3.79 | 52 |
| TP | Tuopaishede | Sichuan Suining | 3.75 | 52 |
| GJG | Gujinggong | Anhui Bozhou | 3.78 | 50 |
| YH | Yanghe | Jiangsu Suqian | 3.85 | 52 |

**
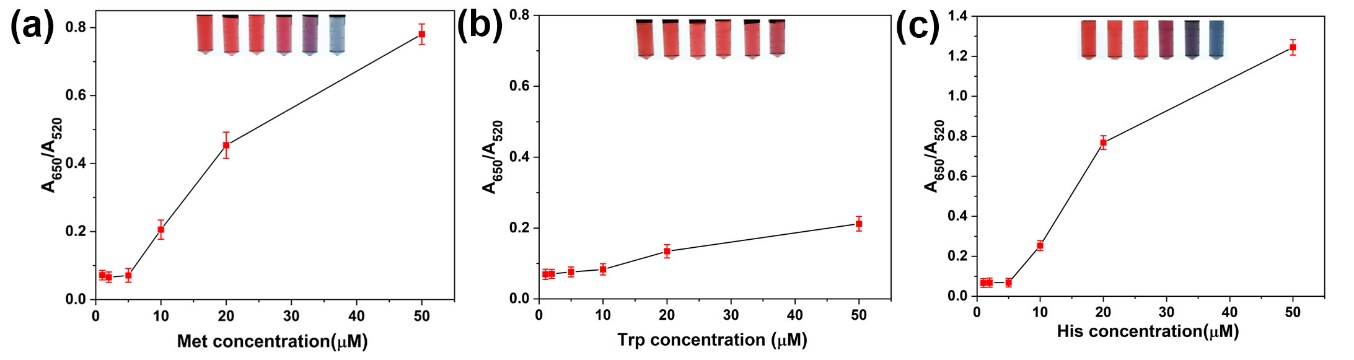
**

**Figure S1.** Optimization of concentration of amino acid modified on the surface of AuNPs.

**
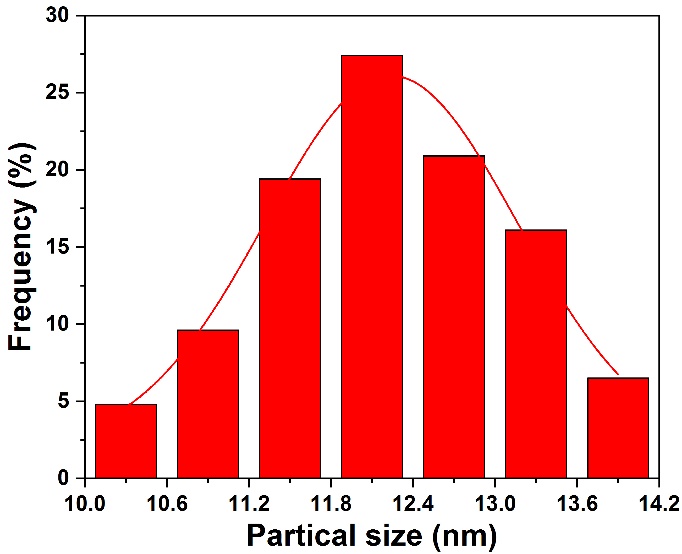
**

**Figure S2.** The graph of the Cit@AuNPs DLS analysis.

**
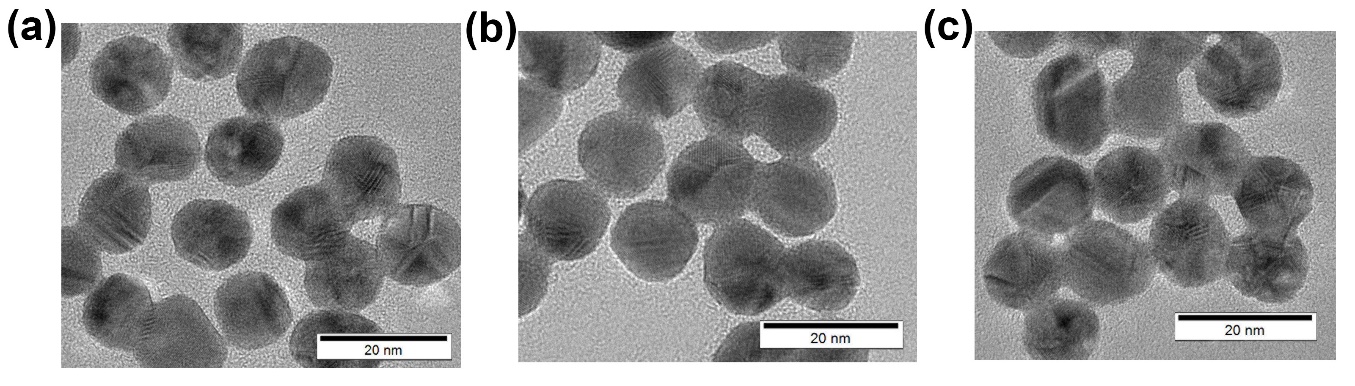
**

**Figure S3.** The TEM of (a) Met@AuNPs; (b) Trp@AuNPs; (c) His@AuNPs.

**
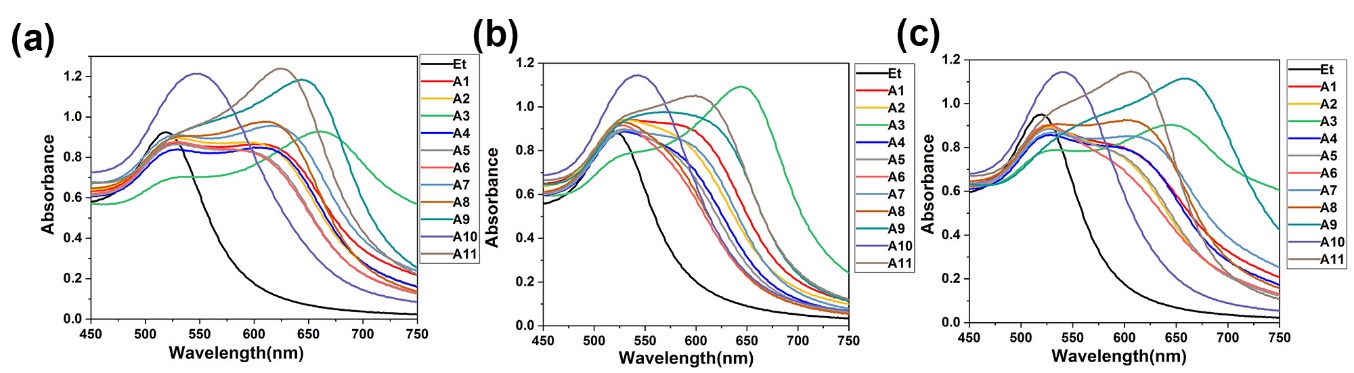
**

**Figure S4.** UV-vis absorption spectra of AA@AuNPs responsed to the organic acids. (a) Met@AuNPs; (b) Trp@AuNPs; (c) His@AuNPs.


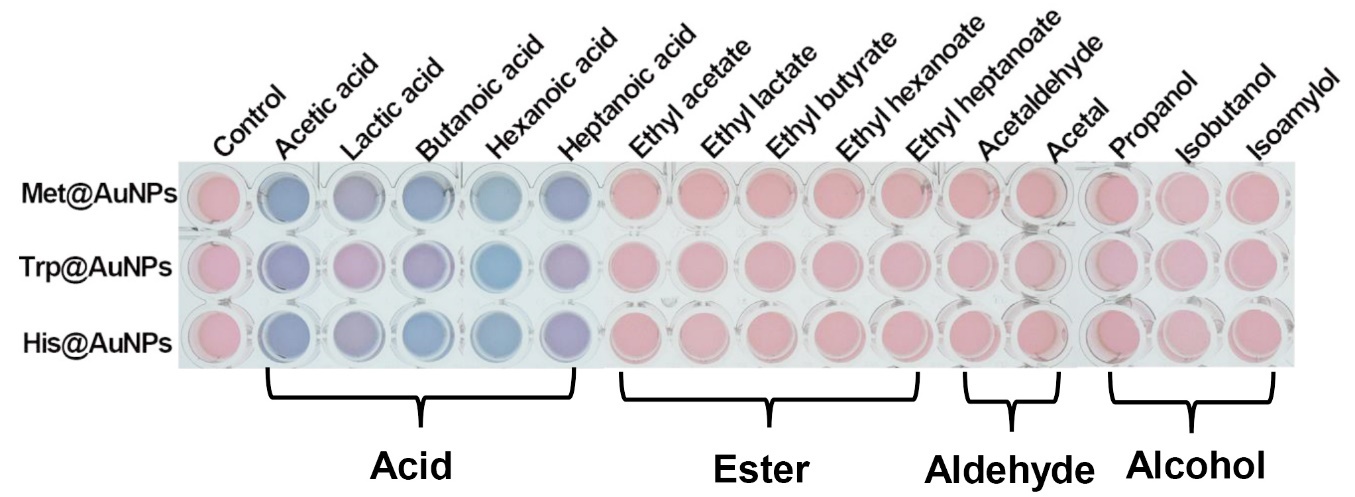


**Figure S5.** Response of the array based on AA@AuNPs to mainly flavor compounds in baijiu.


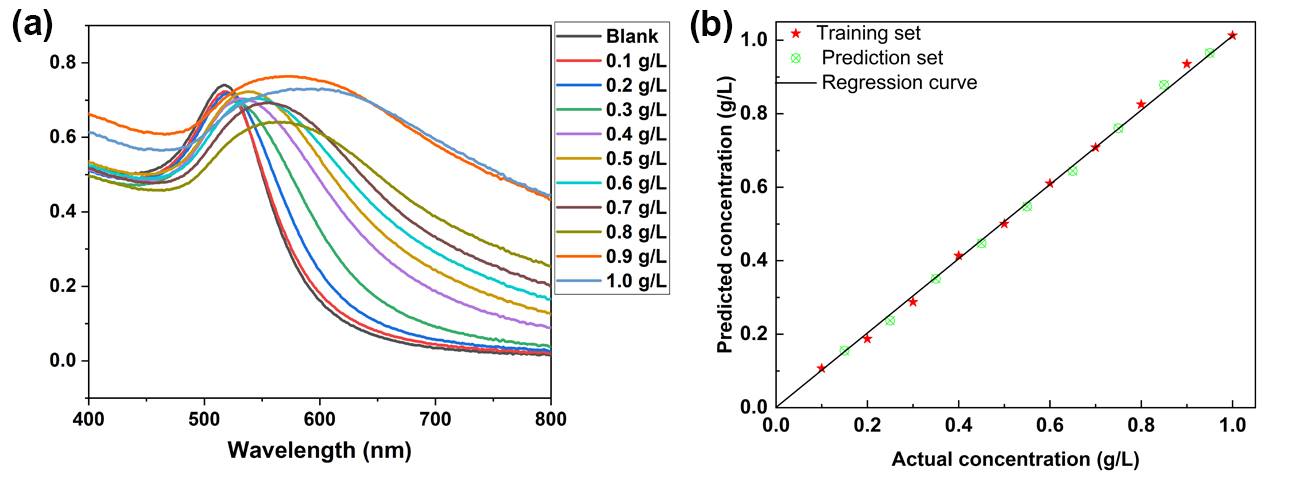


**Figure S6.**  (a) UV-vis spectrum of His@AuNPs response to heptanoic acid of different concentrations; (b) The PLSR model of the three AA@AuNPs response to heptanoic acid of different concentrations.

Table S2. The results of quantitative analysis of heptanoic acid

|  | R^2^ | RMSE (g/L) | RT (%) |
| --- | --- | --- | --- |
| Training set | 0.9754 | 0.0026066 | 103.31±3.49 |
| Prediction set | 0.9988 | 0.0047968 | 101.14±2.93 |

RT: recovery rate; RMSE: root mean square error; R^2^: correlation coefficients


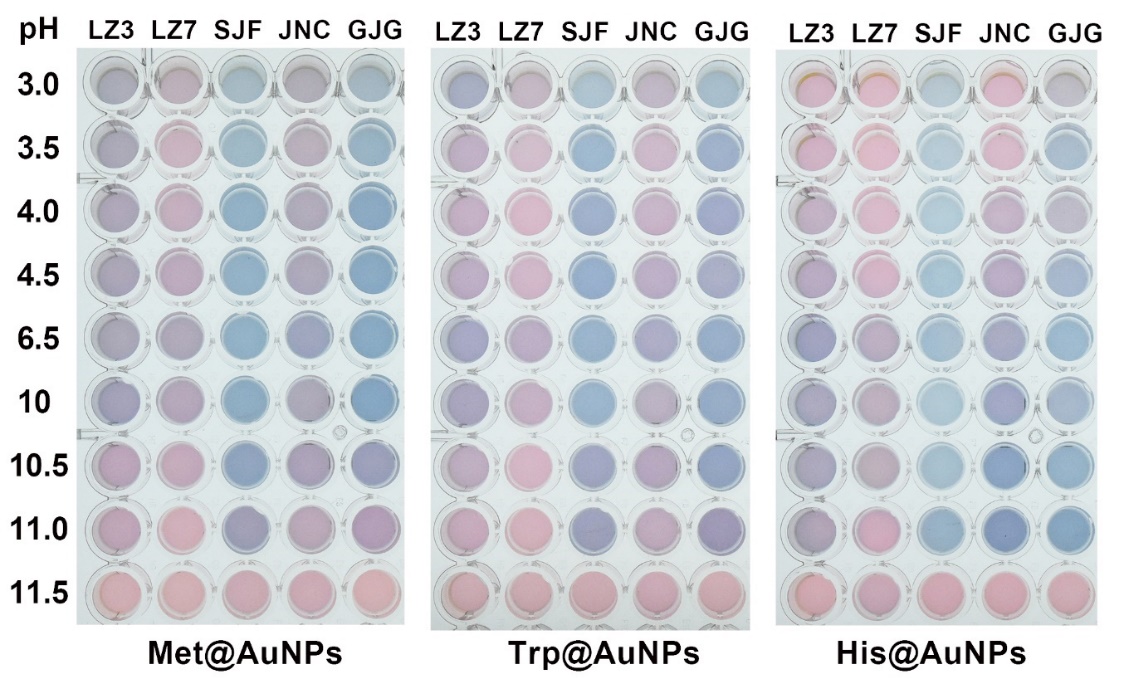


**Figure S7.** Response of AA@AuNPs to five representative SABs under different pH conditions.


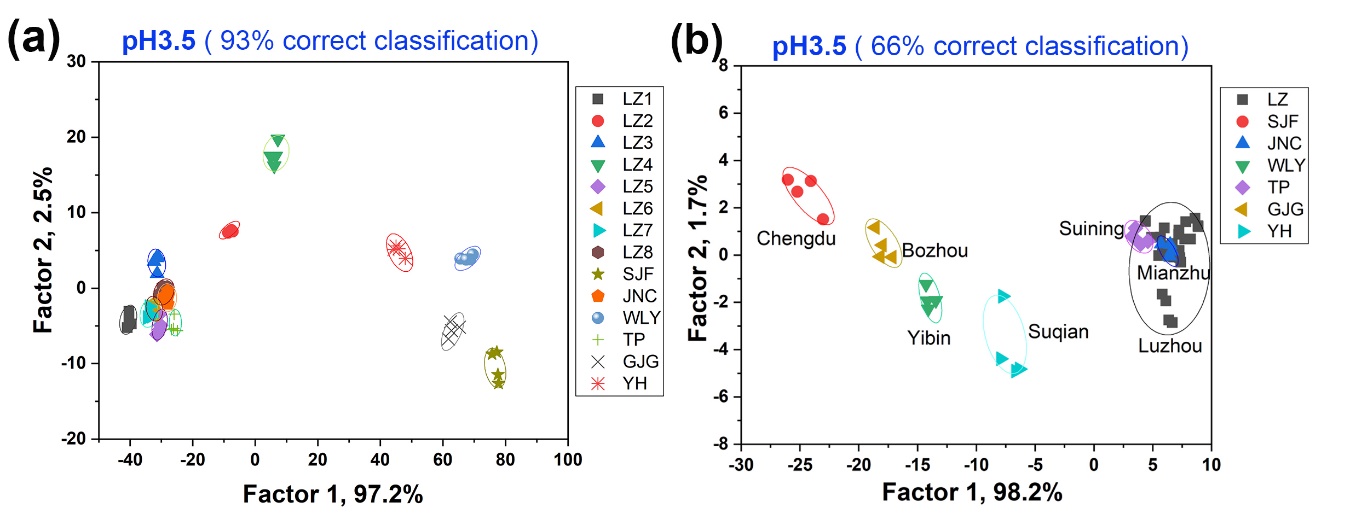


**Figure S8.** Two-dimensional canonical score plots for the first two factors of (a) 14 brands and (b) 7 origins obtained using the sensor array based on AA@AuNPs at the pH 3.5.


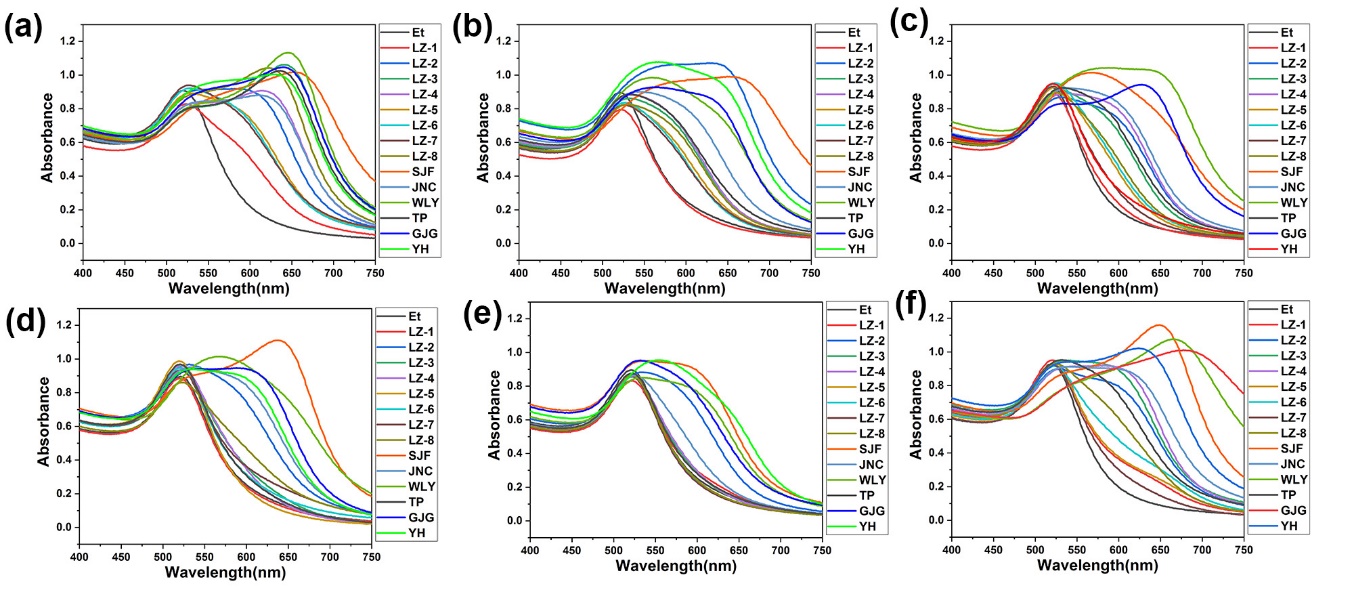


Figure S9. UV-vis absorption spectra of AA@AuNPs responses to different SABs under the two pH conditions. (a) Met @AuNPs at pH 6.5; (b) Trp@AuNPs at pH 6.5; (c) His @AuNPs at pH 6.5; (d) Met @AuNPs at pH 10.5; (e) Trp @AuNPs at pH 10.5; (f) His @AuNPs at pH 10.5.

**Table S3** Jackknifed Classification Matrix of SABs from different brands at pH 6.5

|  | GJG | JNC | LZ-1 | LZ-2 | LZ-3 | LZ-4 | LZ-5 | LZ-6 | LZ-7 | LZ-8 | SJF | TP | WLY | YH | %correct |
| --- | --- | --- | --- | --- | --- | --- | --- | --- | --- | --- | --- | --- | --- | --- | --- |
| **GJG** | 4 | 0 | 0 | 0 | 0 | 0 | 0 | 0 | 0 | 0 | 0 | 0 | 0 | 0 | 100 |
| **JNC** | 0 | 4 | 0 | 0 | 0 | 0 | 0 | 0 | 0 | 0 | 0 | 0 | 0 | 0 | 100 |
| **LZ-1** | 0 | 0 | 4 | 0 | 0 | 0 | 0 | 0 | 0 | 0 | 0 | 0 | 0 | 0 | 100 |
| **LZ-2** | 0 | 0 | 0 | 4 | 0 | 0 | 0 | 0 | 0 | 0 | 0 | 0 | 0 | 0 | 100 |
| **LZ-3** | 0 | 0 | 0 | 0 | 4 | 0 | 0 | 0 | 0 | 0 | 0 | 0 | 0 | 0 | 100 |
| **LZ-4** | 0 | 0 | 0 | 0 | 0 | 4 | 0 | 0 | 0 | 0 | 0 | 0 | 0 | 0 | 100 |
| **LZ-5** | 0 | 0 | 0 | 0 | 0 | 0 | 3 | 0 | 1 | 0 | 0 | 0 | 0 | 0 | 75 |
| **LZ-6** | 0 | 0 | 0 | 0 | 0 | 0 | 0 | 4 | 0 | 0 | 0 | 0 | 0 | 0 | 100 |
| **LZ-7** | 0 | 0 | 0 | 0 | 0 | 0 | 1 | 0 | 3 | 0 | 0 | 0 | 0 | 0 | 75 |
| **LZ-8** | 0 | 0 | 0 | 0 | 0 | 0 | 0 | 0 | 0 | 4 | 0 | 0 | 0 | 0 | 100 |
| **SJF** | 0 | 0 | 0 | 0 | 0 | 0 | 0 | 0 | 0 | 0 | 4 | 0 | 0 | 0 | 100 |
| **TP** | 0 | 0 | 0 | 0 | 0 | 0 | 0 | 0 | 0 | 0 | 0 | 4 | 0 | 0 | 100 |
| **WLY** | 0 | 0 | 0 | 0 | 0 | 0 | 0 | 0 | 0 | 0 | 0 | 0 | 4 | 0 | 100 |
| **YH** | 0 | 0 | 0 | 0 | 0 | 0 | 0 | 0 | 0 | 0 | 0 | 0 | 0 | 4 | 100 |
| **Total** | 4 | 4 | 4 | 4 | 4 | 4 | 4 | 4 | 4 | 4 | 4 | 4 | 4 | 4 | 96 |

**Figure S10**. Correlations of canonical colorimetric response patterns from AA@AuNPs against the different brands of SABs at pH 6.5.

**Table S4** Jackknifed Classification Matrix of SABs from different brands at pH10.5

|  | GJG | JNC | LZ-1 | LZ-2 | LZ-3 | LZ-4 | LZ-5 | LZ-6 | LZ-7 | LZ-8 | SJF | TP | WLY | YH | %correct |
| --- | --- | --- | --- | --- | --- | --- | --- | --- | --- | --- | --- | --- | --- | --- | --- |
| **GJG** | 4 | 0 | 0 | 0 | 0 | 0 | 0 | 0 | 0 | 0 | 0 | 0 | 0 | 0 | 100 |
| **JNC** | 0 | 4 | 0 | 0 | 0 | 0 | 0 | 0 | 0 | 0 | 0 | 0 | 0 | 0 | 100 |
| **LZ-1** | 0 | 0 | 4 | 0 | 0 | 0 | 0 | 0 | 0 | 0 | 0 | 0 | 0 | 0 | 100 |
| **LZ-2** | 0 | 0 | 0 | 4 | 0 | 0 | 0 | 0 | 0 | 0 | 0 | 0 | 0 | 0 | 100 |
| **LZ-3** | 0 | 0 | 0 | 0 | 4 | 0 | 0 | 0 | 0 | 0 | 0 | 0 | 0 | 0 | 100 |
| **LZ-4** | 0 | 0 | 0 | 0 | 0 | 4 | 0 | 0 | 0 | 0 | 0 | 0 | 0 | 0 | 100 |
| **LZ-5** | 0 | 0 | 0 | 0 | 0 | 0 | 4 | 0 | 0 | 0 | 0 | 0 | 0 | 0 | 100 |
| **LZ-6** | 0 | 0 | 0 | 0 | 0 | 0 | 0 | 4 | 0 | 0 | 0 | 0 | 0 | 0 | 100 |
| **LZ-7** | 0 | 0 | 0 | 0 | 0 | 0 | 0 | 0 | 4 | 0 | 0 | 0 | 0 | 0 | 100 |
| **LZ-8** | 0 | 0 | 0 | 0 | 0 | 0 | 0 | 0 | 0 | 4 | 0 | 0 | 0 | 0 | 100 |
| **SJF** | 0 | 0 | 0 | 0 | 0 | 0 | 0 | 0 | 0 | 0 | 4 | 0 | 0 | 0 | 100 |
| **TP** | 0 | 0 | 0 | 0 | 0 | 0 | 0 | 0 | 0 | 0 | 0 | 4 | 0 | 0 | 100 |
| **WLY** | 0 | 0 | 0 | 0 | 0 | 0 | 0 | 0 | 0 | 0 | 0 | 0 | 4 | 0 | 100 |
| **YH** | 0 | 0 | 0 | 0 | 0 | 0 | 0 | 0 | 0 | 0 | 0 | 0 | 0 | 4 | 100 |
| **Total** | 4 | 4 | 4 | 4 | 4 | 4 | 4 | 4 | 4 | 4 | 4 | 4 | 4 | 4 | 100 |

**Figure S11.** Correlations of canonical colorimetric response patterns from AA@AuNPs against the different brands of SABs at pH10.5.

**Table S5** Jackknifed Classification Matrix of SABs from different brands at pH 6.5 and pH10.5

|  | GJG | JNC | LZ-1 | LZ-2 | LZ-3 | LZ-4 | LZ-5 | LZ-6 | LZ-7 | LZ-8 | SJF | TP | WLY | YH | %correct |
| --- | --- | --- | --- | --- | --- | --- | --- | --- | --- | --- | --- | --- | --- | --- | --- |
| **GJG** | 4 | 0 | 0 | 0 | 0 | 0 | 0 | 0 | 0 | 0 | 0 | 0 | 0 | 0 | 100 |
| **JNC** | 0 | 4 | 0 | 0 | 0 | 0 | 0 | 0 | 0 | 0 | 0 | 0 | 0 | 0 | 100 |
| **LZ-1** | 0 | 0 | 4 | 0 | 0 | 0 | 0 | 0 | 0 | 0 | 0 | 0 | 0 | 0 | 100 |
| **LZ-2** | 0 | 0 | 0 | 4 | 0 | 0 | 0 | 0 | 0 | 0 | 0 | 0 | 0 | 0 | 100 |
| **LZ-3** | 0 | 0 | 0 | 0 | 4 | 0 | 0 | 0 | 0 | 0 | 0 | 0 | 0 | 0 | 100 |
| **LZ-4** | 0 | 0 | 0 | 0 | 0 | 4 | 0 | 0 | 0 | 0 | 0 | 0 | 0 | 0 | 100 |
| **LZ-5** | 0 | 0 | 0 | 0 | 0 | 0 | 4 | 0 | 0 | 0 | 0 | 0 | 0 | 0 | 100 |
| **LZ-6** | 0 | 0 | 0 | 0 | 0 | 0 | 0 | 4 | 0 | 0 | 0 | 0 | 0 | 0 | 100 |
| **LZ-7** | 0 | 0 | 0 | 0 | 0 | 0 | 0 | 0 | 4 | 0 | 0 | 0 | 0 | 0 | 100 |
| **LZ-8** | 0 | 0 | 0 | 0 | 0 | 0 | 0 | 0 | 0 | 4 | 0 | 0 | 0 | 0 | 100 |
| **SJF** | 0 | 0 | 0 | 0 | 0 | 0 | 0 | 0 | 0 | 0 | 4 | 0 | 0 | 0 | 100 |
| **TP** | 0 | 0 | 0 | 0 | 0 | 0 | 0 | 0 | 0 | 0 | 0 | 4 | 0 | 0 | 100 |
| **WLY** | 0 | 0 | 0 | 0 | 0 | 0 | 0 | 0 | 0 | 0 | 0 | 0 | 4 | 0 | 100 |
| **YH** | 0 | 0 | 0 | 0 | 0 | 0 | 0 | 0 | 0 | 0 | 0 | 0 | 0 | 4 | 100 |
| **Total** | 4 | 4 | 4 | 4 | 4 | 4 | 4 | 4 | 4 | 4 | 4 | 4 | 4 | 4 | 100 |

**Figure S12.** Correlations of canonical colorimetric response patterns from AA@AuNPs against the different brands of SABs at pH 6.5 and pH10.5.

**Table S6** Jackknifed Classification Matrix of SABs from different origins at pH 6.5

|  | GJG | JNC | LZ | SJF | TP | WLY | YH | %correct |
| --- | --- | --- | --- | --- | --- | --- | --- | --- |
| **GJG** | 4 | 0 | 0 | 0 | 0 | 0 | 0 | 100 |
| **JNC** | 0 | 4 | 0 | 0 | 0 | 0 | 0 | 100 |
| **LZ** | 0 | 4 | 27 | 0 | 1 | 0 | 0 | 84 |
| **SJF** | 0 | 0 | 0 | 4 | 0 | 0 | 0 | 100 |
| **TP** | 0 | 0 | 0 | 0 | 4 | 0 | 0 | 100 |
| **WLY** | 0 | 0 | 0 | 0 | 0 | 4 | 0 | 100 |
| **YH** | 0 | 0 | 0 | 0 | 0 | 0 | 4 | 100 |
| **Total** | 4 | 8 | 27 | 4 | 5 | 4 | 4 | 91 |

**Figure S13.** Correlations of canonical colorimetric response patterns from AA@AuNPs against the different brands of SABs at pH 6.5.

**Table S7** Jackknifed Classification Matrix of SABs from different origins at pH10.5

|  | GJG | JNC | LZ | SJF | TP | WLY | YH | %correct |
| --- | --- | --- | --- | --- | --- | --- | --- | --- |
| **GJG** | 4 | 0 | 0 | 0 | 0 | 0 | 0 | 100 |
| **JNC** | 0 | 4 | 0 | 0 | 0 | 0 | 0 | 100 |
| **LZ** | 0 | 0 | 16 | 0 | 16 | 0 | 0 | 50 |
| **SJF** | 0 | 0 | 0 | 4 | 0 | 0 | 0 | 100 |
| **TP** | 0 | 0 | 0 | 0 | 4 | 0 | 0 | 100 |
| **WLY** | 0 | 0 | 0 | 0 | 0 | 4 | 0 | 100 |
| **YH** | 0 | 0 | 0 | 0 | 0 | 0 | 4 | 100 |
| **Total** | 4 | 4 | 16 | 4 | 20 | 4 | 4 | 71 |

**Figure S14.** Correlations of canonical colorimetric response patterns from AA@AuNPs against the different brands of SABs at pH10.5.

**Table S8** Jackknifed Classification Matrix of SABs from different origins at pH 6.5 and pH10.5

|  | GJG | JNC | LZ | SJF | TP | WLY | YH | % correct |
| --- | --- | --- | --- | --- | --- | --- | --- | --- |
| **GJG** | 4 | 0 | 0 | 0 | 0 | 0 | 0 | 100 |
| **JNC** | 0 | 4 | 0 | 0 | 0 | 0 | 0 | 100 |
| **LZ** | 0 | 1 | 31 | 0 | 0 | 0 | 0 | 97 |
| **SJF** | 0 | 0 | 0 | 4 | 0 | 0 | 0 | 100 |
| **TP** | 0 | 0 | 0 | 0 | 4 | 0 | 0 | 100 |
| **WLY** | 0 | 0 | 0 | 0 | 0 | 4 | 0 | 100 |
| **YH** | 0 | 0 | 0 | 0 | 0 | 0 | 4 | 100 |
| **Total** | 4 | 5 | 31 | 4 | 4 | 4 | 4 | 98 |

**Figure S15.** Correlations of canonical colorimetric response patterns from AA@AuNPs against the different brands of SABs at pH 6.5 and pH10.5.
